# Instance-level 6D pose estimation based on multi-task parameter sharing for robotic grasping

**DOI:** 10.1038/s41598-024-58590-x

**Published:** 2024-04-02

**Authors:** Liming Zhang, Xin Zhou, Jiaqing Liu, Can Wang, Xinyu Wu

**Affiliations:** 1 Guangdong Provincial Key Lab of Robotics and Intelligent System, Shenzhen Institute of Advanced Technology, Chinese Academy of Sciences, Shenzhen, 518055 China; 2 University of Science and Technology of China, Hefei, 230026 China

**Keywords:** Computer science, Information technology

## Abstract

Six-dimensional pose estimation task predicts its 3D rotation matrix and 3D translation matrix in the world coordinate system by inputting the color image or depth image of the target object. Existing methods usually use deep neural networks to directly predict or regress object poses based on keypoint methods. The prediction results usually have deviations depending on whether the surface shape of the object is prominent or not and the size of the object. To solve this problem, we propose the six-dimensional pose estimation based on multi-task parameter sharing (PMP) framework to incorporate object category information into the pose estimation network through the form of an object classification auxiliary task. First, we extract the image features and point cloud features of the target object separately, and fuse them point by point; then, we share the confidence of each keypoint in pose estimation task and the knowledge of the classification task, get the key points with higher confidence, and predict the object pose; finally, the obtained object pose is passed through an iterative optimization network to obtain the final pose. The experimental results on the LineMOD dataset show that the proposed method can improve the accuracy of pose estimation and narrow the gap in the prediction accuracy of objects with different shapes. We also tested on a new dataset of small-scale objects, which contains object RGBD images and accurate 3D point cloud information. The proposed method is applied to the grasping experiment on the UR5 robotic arm, which satisfies the real-time pose estimation results during the grasping process.

## Introduction

Six-dimensional pose estimation has various applications in robotic arm grasping, augmented reality, and autonomous driving. The most commonly used algorithms in pose estimation are mainly based on RGB images, depth images or multimodal signals that fuse RGB images and depth images, and then common deep neural networks are used for prediction, which can be divided into direct prediction methods, keypoint-based methods, and iterative optimization methods. In direct prediction technology, existing methods use deep neural networks to directly extract the features of the input data for end-to-end prediction. In keypoint-based technology, Peng et al.^1^ proposed a network PVNet to deal with object occlusion in 2019, which proposed to use the voting method to determine the keypoints of the object. Rad et al.^2^ proposed BB8 to divide input RGB images into coarse and fine-grained image blocks that were fed into a convolutional classification network to predict the 2D projection coordinates corresponding to the 3D enclosing frame of the target object. Compared with the direct methods, the keypoint-based methods find the keypoints of the object to be recognized and establishes a 2D–3D correspondence, which is more conducive to the pose estimation of the object. Based on the previous two methods, the purpose of iterative optimization network is to further optimize the predicted pose estimation results, which can be divided into keypoint registration method and neural network optimization method. Besl et al.^3^ proposed Iterative Closest Point (ICP) to continuously iterate the nearest point and find the optimal transformation, which can get better registration results. However, this method consumes a large amount of computing resources and takes a long time, Wang et al.^4^ proposed DenseFusion to replace the iterative closest point algorithm with a deep learning network, which can be jointly optimized with the overall network for end-to-end pose estimation.

Nevertheless, only improving the feature extraction capability is insufficient for the pose-estimation problem. Notably, six-dimensional pose estimation method suffers from the following problems. On the one hand, the way it predicts makes it less interpretable. In practice, the pose-estimation problem must obtain the predicted 3D model by predicting translation vectors and rotation matrices. As a result, without any prior knowledge, it must continue reducing the distance between the 3D predicted model and the 3D ground truth model, while the real rotation and translation matrices are not exposed to the method. On the other hand, six-dimensional pose estimation results usually vary with the prominence and size of the object’s surface. For large-scale objects and small-scale objects or objects with low surface features, the estimation results usually have large deviations.

According to existing multi-tasking techniques^5,6^, complex problems can be solved by decomposing into multiple sub-problems. Zhou et al.^5^ reported that the complex problem can first be refined and decomposed into simple and mutually independent sub-problems. These sub-problems are connected and interconnected by common characteristics, which can help the primary task pick up new features easily and strengthen generalization capability. Vandenhende et al.^6^ highlighted that numerous issues are intricate and require multiple steps to resolve. For example, the problem of determining the safety of a self-driving car can usually be broken down into the following steps: Identify objects in the scene, detect and localize them, and estimate the motion trajectory. The parameter-sharing mechanism in multi-tasking techniques could be divided into soft-sharing, hard-sharing, and layer-sharing. Soft-sharing^7^ methods make each task have its own parameters, encouraging parameters to be more similar through regularization. Compared with soft-sharing, hard-sharing^8–10^ methods can greatly reduce the number of parameters, which use shared layers aimed at sharing hidden parameters and apply a task-specific layer for each task. For example, to train jointly for numerous tasks, Sun et al.^11^ proposed a hard-sharing method called sparse sharing, in order to abstract more similar sub-networks with higher overlap for strongly linked tasks and less overlapping sub-networks with larger differences for weakly related tasks, they experimentally demonstrated it requires the least number of parameters and exhibited superior accuracy in multi-task natural language interpretation.

Inspired by the concept of sparse sharing^11^, we modeled the multi-task approach to jointly optimize the object classification task and pose estimation task. Specifically, the object classification task is applied as an auxiliary task to provide inductive knowledge for pose estimation. Our contributions can be summarized as follows:

- We have created a multi-angle dataset featuring small-sized objects. PMP dataset was designed to address the performance limitations observed in existing methods when dealing with small objects in the LineMOD dataset. The dataset includes images capturing various postures of the objects, simulating the states observed during robotic arm gripping.
- We proposed a PMP framework for multi-task pose estimation based on parameter sharing. By employing a parameter sharing approach, object classification is used as an auxiliary task to provide object category information for keypoint feature extraction in pose estimation. This helps reduce prediction biases among different-shaped objects and improves accuracy.
- We not only achieves higher accuracy but also exhibits smaller accuracy deviations among objects than existing pose estimating models on the LineMOD dataset. Even in robotic grasping scenarios, the PMP model effectively estimates the pose of the objects.

The remainder of our paper is structured as follows. Section II describes the overall architecture of the our method and the details of each part of the implementation, Section III describes the dataset and associated experimental results. Finally, Section IV discusses the contributions of the model and prospects.

**Figure 1:**
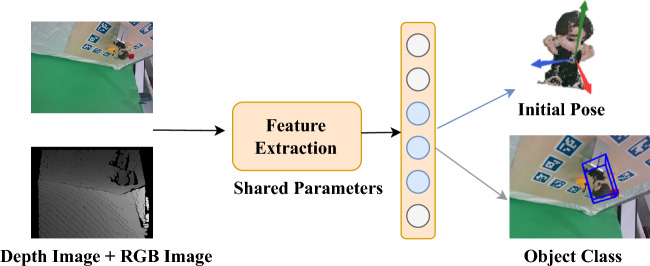
Pipeline of our method. The pose-estimation task in the figure is performed using the blue neurons, whereas the target object categorization task is performed using the gray neurons. The accuracy of posture estimate and the ability of the model to generalize can both be increased as the two associated tasks can share and supplement each other’s learned knowledge.

## Methodology

Given an RGBD image, the goal of 6D pose estimation is to obtain the translation vector $T \in \mathbb {R}^{3}$ and rotation matrix $R \in S O(3)$ of the target of interest from the world coordinate system to the camera coordinate system. The prediction results usually have deviations depending on whether the surface shape of the object is prominent or not and the size of the object. To solve this problem, a novel method was proposed herein. As shown in Fig. 1, we applied multi-tasking techniques for this problem, and the target object poses and categories are predicted by randomly discarding different neurons.

**Figure 2:**
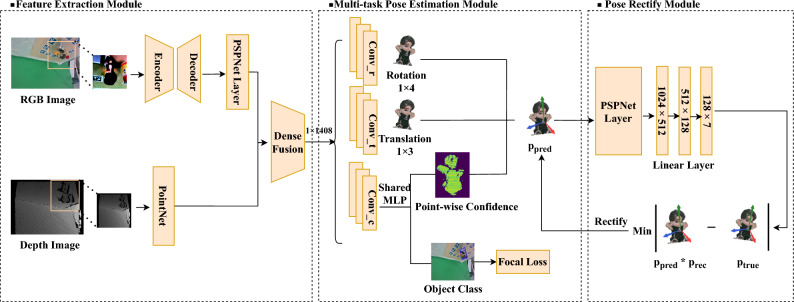
Overall structure of our method. The model structure is divided into three modules, the feature extraction module, multi-task pose-estimation module, and pose rectify module.

The method consists of three parts. As shown in Fig. 2, the first part extracts and fuses the RGB and depth features of the target object. The second part comprises object pose estimation and classification through shared network parameters. The third part refines and recalculates the object pose to obtain the pose result more precisely.

### Feature extraction module

#### Image feature extraction

The image feature extraction module is split into two sections that are based on a transformer^12^ and CNN^13^. First, the input image crops are cut into $size_{p} \times size_{p}$ image patches, and then each image patch is encoded into one-dimensional features, that are fed into the transformer-based encoder-decoder structure. The encoder extracts the high dimensional global hidden features of the input image features, then a lightweight decoder reduces the extracted hidden features to the original image size such that each pixel point contains global attention to the pixels in the image. Compared with the symmetric encoder-decoder, the asymmetric structure uses a lighter-weight decoder, which greatly reduces the amount of calculation and hides more high-dimensional information in the encoder.

The reduced global features of the decoder are then sent into a multi-scale convolution structure PSPNet^14^ layer, the multi-scale convolutional network can use its inductive bias to extract local information, it uses a pyramid structure of multi-scale feature fusion to enhance the attention to local pixels around the pixel point.

**Figure 3:**
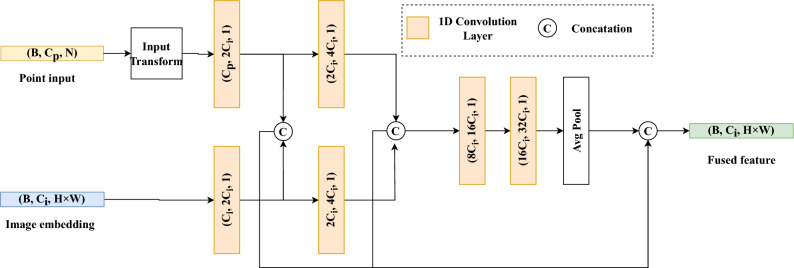
The process of point cloud feature extraction and feature fusion. Point cloud features are extracted using the Input Transform Layer, multiple-scale convolutional layers, and average pooling.

#### Point cloud feature extraction

Fig. 3 illustrates the process of point cloud feature extraction and fusion with image features. The global features of the point cloud data are extracted through the Input Transform Layer, multiple-scale convolutional layers, and average pooling operations. The input transform layer, originally introduced in PointNet^15^, introduces permutation invariance to the model by multiplying the point cloud data with a $C{_p}$ $\times$ $C{_p}$ transformation matrix, making it insensitive to the order of points. Multiple-scale convolutional layers support the extraction of multi-scale features from the point cloud and enable dense fusion with image features. In Fig. 3, $C{_i}$ represents the feature output channels in the image feature extraction module, $C{_p}$ represents the channels of the input point cloud, and the output of the dense fusion network is the concatenation of features from multiple scales.

### Multi-task pose-estimation module

**Figure 4:**
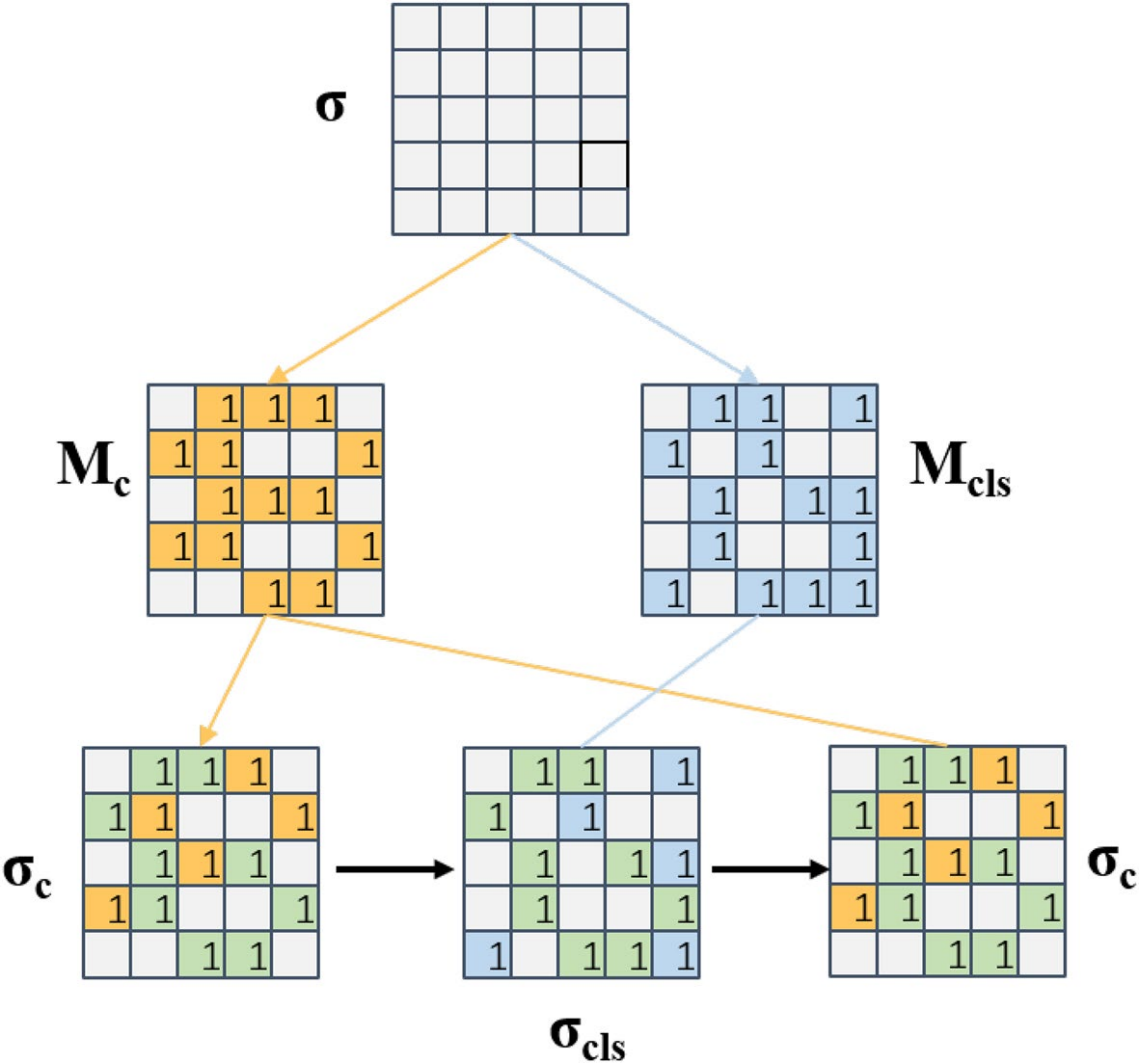
Schematic of the shared parameters between the pose estimation and classification task. The orange and blue parts of the diagram represent various parts of various sub-networks, whereas the green parts of the diagram indicate the overlapping parts of the sub-networks.

After extracting and fusing the RGB and depth feature of the input image crops, the next step is to determine the key point information of the target object. For the extracted multimodal features, three separate convolutional neural networks are used to predict the 6D pose $p=[R\mid T]$ of the target object, which contains the translation vector $T \in \mathbb {R}^{3}$ and rotation matrix $R \in S O(3)$, while the third network is used to predict the key point confidence *c* for pose estimation task and object class information *cls* for classification task simultaneously, which is completed in the form of shared network parameters. Conv_t, Conv_r, and Conv_c in Fig. 2 are all one-dimensional convolutions that are fed separately with the per-point feature vectors that generated from the feature extraction module. They are mapped using the translation, rotation, and confidence vectors into features with dimensions of num_obj*4, num_obj*3, and num_obj*1, respectively, where num_obj represents the total number of objects in the current dataset.

Inspired by Sun et al.^11^, we utilized a shared neural network with the purpose of adding classification task as an auxiliary task for the six-dimensional pose estimation, and it is expected to provide prior Knowledge. Three concepts are involved here: base network, subnetwork, and shared network. The base network refers to the network that contains all the parameters of the model, and the sub-network is a part extracted from the base network, and the common part of all sub-networks is the shared network. As shown in Fig. 4, $\sigma$ denotes the base network. To generate key point confidence while predicting object categories, and to simultaneously enhance the confidence credibility and persuasiveness, different masks $M_{c}$ and $M_{cls}$, were generated and sub-networks $\sigma _{c}$ and $\sigma _{cls}$ with both difference and commonality from the base network were extracted for the two tasks; subsequently, the two tasks were trained in parallel. As a result, the base network $\sigma$ can contain solutions for multiple tasks such that the two associated tasks can share the learned knowledge through the common sub-network, and the negative transfer due to differences in tasks can be avoided through the differences in the sub-network.

For the pose-estimation task, as in the previous work^16^, ADD was used to calculate the difference in distance between the corresponding points of the 3D model of the object transformed by the ground truth pose $p=[R \mid T]$ and the pose $\hat{p}_{pred}=\left[ \hat{R}_{i} \mid \hat{T_{i}}\right]$ obtained from the model estimation. Thus, the accuracy of the object pose estimation is:

1 $$\begin{aligned} D_{i}^{p}=\frac{1}{m} \sum _{j \in M}\left\| \left( R x_{j}+T\right) -\left( \hat{R}_{i} x_{j}+\hat{T}_{i}\right) \right\| , \end{aligned}$$

where *i* indicates the *i* *th* key point, $D_{i}^{p}$ represents the mean distance between point pairs onset by the *i* *th* dense-pixel prediction result $\hat{p_{i}}=\left[ \hat{R}_{i} \mid \hat{T_{i}}\right]$, $x_{j}$ represents the *j* *th* 3D point among the selected 3D point clouds set *M*, and *m* is the number of *M*.

However, a symmetric object representation has multiple pose-estimation results $\hat{p}_{pred}=\left[ \hat{R}_{i} \mid \hat{T_{i}}\right]$ that all achieve the same shape as the ground truth poses $p=[R \mid T]$ and cannot be measured using the strict computational method ADD. The ADD-S proposed in PoseCNN^17^ determines the distance between the 3D model predicted by the target object and the nearest point of the ground truth 3D model. Herein, it was used to measure the accuracy of the positional estimation of the symmetric object, as follows.

2 $$\begin{aligned} D_{i}^{p}=\frac{1}{m} \sum _{j,k \in M} \min _{0<k<m}\left\| \left( R x_{j}+T\right) -\left( \hat{R}_{i} x_{k}+\hat{T}_{i}\right) \right\| , \end{aligned}$$

where $x_{k}$ represents the point with the smallest distance difference from the current point $x_{j}$ after pose conversion.

For multiple point predictions of the poses of the object $p_{i}=\{R|T\}_{i}, i \in [1, N]$, where *N* represents the number of key points in the object, the confidence level *c* was employed to set the pose loss:

3 $$\begin{aligned} L_{p}=\frac{1}{n} \sum _{i \in N}\left( D_{i}^{p} c_{i}-w \log \left( c_{i}\right) \right) , \end{aligned}$$

where *i* denotes the *i* *th* key point, *N* is the number of key points, $c_{i}$ is the confidence of the predicted poses of the key points, and $D_{i}^{p}$ denotes the distance between the predicted and true poses of each key point, which was calculated using ADD^16^ and ADD-S^17^ for symmetric and asymmetric objects, respectively. To balance the distance and confidence, and avoid $c_{i}$ being excessively small, weight *w* was added to limit the size of the confidence $c_{i}$.

For the classification task, as individual objects in the dataset vary in shape and object size, focal loss^18^ was used to overcome the sample imbalance and difficult sample mining problem by using it to predict the object class *cls*:

4 $$\begin{aligned} L_{cls}=-\left( 1-p_{\textrm{cls}}\right) ^\gamma \log \left( p_{\textrm{cls}}\right) , \end{aligned}$$

where $p_{cls}$ is the probability of the prediction being category *cls*. The $\gamma$ parameter, called a modulating factor, is used to reduce the weight of the easily classified samples to ensure that the model focuses on the hard-to-classify samples.

To supervise two tasks simultaneously, the final multi-task loss is:

5 $$\begin{aligned} L=L_{p}+\lambda L_{cls}, \end{aligned}$$

where $\lambda$ is set to an initial value of $\lambda _{0}$ at initialization; as the training epoch increases, $\lambda$ decreases exponentially according to $2^{-i}$. According to^19^, training the auxiliary tasks first, and sharing the learned knowledge as a “hint” to the main task can improve the generalization ability of the model. The decrease in $\lambda$ in the method allows the model to learn the less difficult target classification; consequently, the model learns more potential feature expressions and the speed of learning pose estimation increases.

### Pose rectify module

When all the above steps are completed, the calculated pose and generated picture feature are sent into the pose rectify module. The primary goal of this step is to fine-tune the roughly approximated pose and bring it closer to the original pose of the object. As shown in Fig. 2, the pose rectify module consists of a PSPNet^14^ layer and a series of consecutive linear layers, and the output of this module is $p_{rec}$, which is a fine-tuning relative to the pose-estimation module. Herein, the pose $p_{pred} \times p_{rec}$ obtained by the two-step adjustment of the object and the ground truth pose $p_{true}$ were used to map the three-dimensional points of the object in the world coordinate system to the counterpart in the camera coordinate system, which were named as $P_{pred}$ and $P_{true}$.

## Experiments

The LineMOD^16^ and PMP datasets were used herein. The LineMOD dataset was first published in the year 2012 and has become a benchmark for 6D pose estimation, while the PMP dataset is a small-sized objects dataset that we captured using UR5 and RealSense D455 cameras. It also contains RGB and depth images, and it is used to verify the accuracy of pose estimation of small-sized objects by our method in the case of real robotic arm grasping. The diameter of the objects in the LineMOD and PMP dataset can be observed from Table 1.

**Table 1:** The diameter of objects in the LineMOD and PMP dataset. The sizes of objects in the LineMOD dataset are displayed by the first three rows, while the sizes of objects in the PMP dataset are displayed by the final row.

Object	Diameter	Object	Diameter	Object	Diameter	Object	Diameter	Object	Diameter
Ape	102.1	Bench vi.	247.5	Camera	172.5	Phone	212.4	Hole p.	145.5
Can	201.4	Cat	154.5	Driller	261.5	Lamp	282.6	Iron	278.1
Duck	109.0	Eggbox	164.6	Glue	175.9				
Boy	139.8	Duck	66.0	Roll	47.2	Shelf	100.8	Glue	112.4

The numbers in the table are in millimeters (mm) as the unit.

Indicates the symmetric objects.

### Datasets

**Figure 5:**
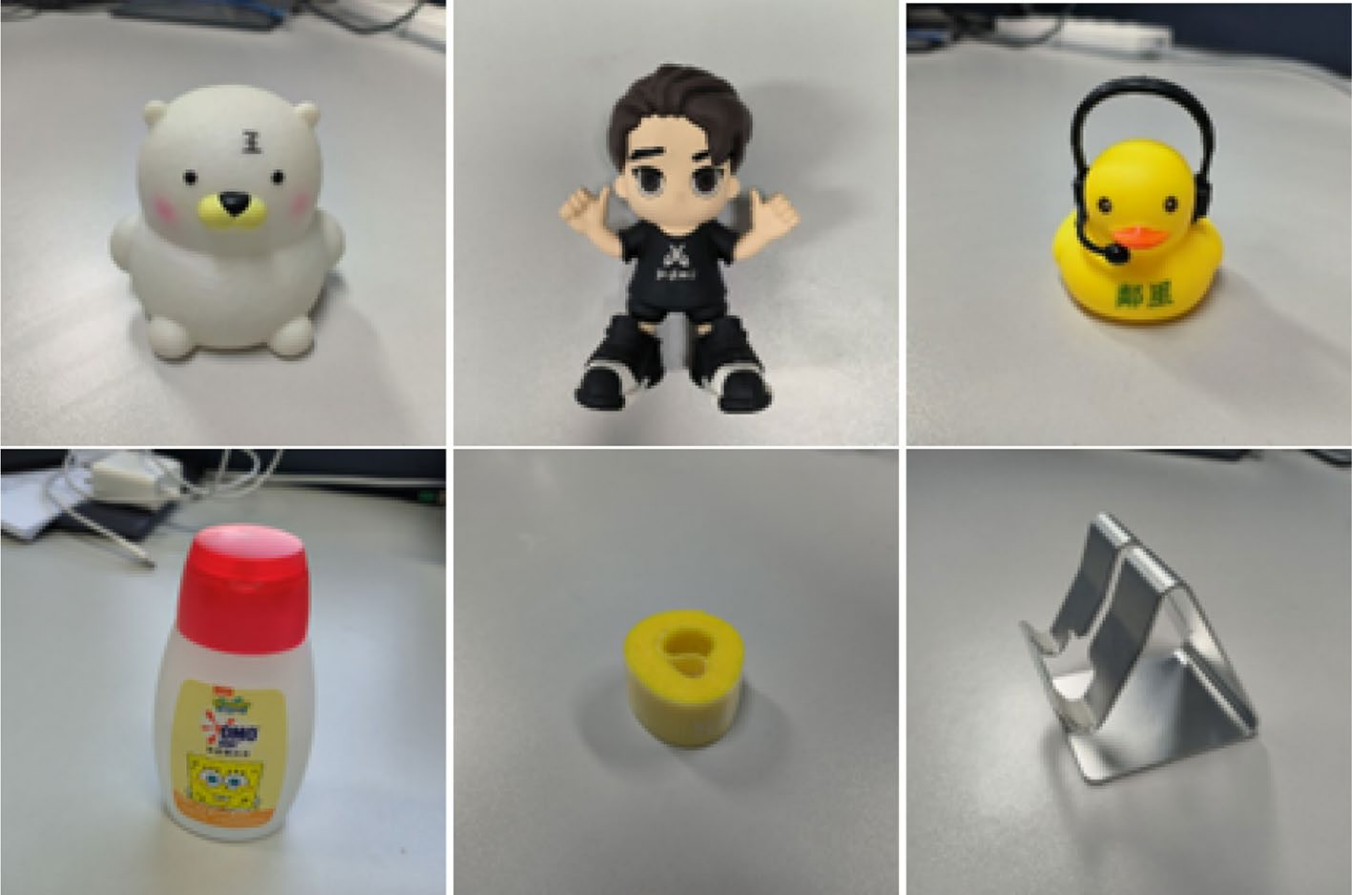
Object display in the PMP dataset.

1. * LineMOD Dataset:* The LineMOD dataset^16^ is widely used in pose estimation. It consists of 13 image sequences, each sequence comprises RGBD images of a single object, with a total of 15 objects, and the 3D point cloud model of each object is provided. Thus, it contains approximately 15,000 images. The minimum diameter of the object is 10.2 cm, and the largest can reach 28.2 cm.
2. *PMP Dataset:* The PMP dataset is an RGBD dataset taken with the RealSense D455 camera and the UR5 robotic arm. Unlike the traditional pose-estimation dataset, it is obtained by shooting 360$^{\circ }$ around the target object, and the poses vary considerably between frames. Each object is shown separately in Fig. 5 below.

It contains five image sequences, each sequence has an average of 1100 images, and the total number of images exceeds 5000. It comprises six objects, namely bear, duck, shelf, glue, roll, and boy. Compared with the LineMOD dataset, the object size is smaller, and the diameter fluctuates between 13.9 cm and 4.7 cm.

### Metrics

The objects in the LineMOD^16^ and PMP dataset can be divided into two categories: symmetrical and asymmetrical objects. An asymmetric object is one whose surface shape does not appear repeatedly, that is, when the object remains static, it can only be represented by one pose. The static state of a symmetrical object may not be a single pose, which affects the performance of the model. As in previous work^4,17,20^, the accuracy of pose estimation was measured using the distance difference computed by ADD^16^ and ADD-S^17^, and the result was deemed correct when the distance difference was less than 10% of the object diameter.

### Parameters

In the encoder–decoder model, the width *W* and height *H* of the image cropping block were set to 40, 80, 120, and 160 in increments of 40, according to the size of the target object. To ensure that the image is divided by integer multiples, each segmented image patch size $size_{p}$ was set to 8. The encoder and decoder were not symmetrical. The encoder encodes the image into a high-dimensional feature vector, whereas the lightweight decoder is responsible for restoring the vector to the pixel size of the image. The vector dimension e_embed_dim in the encoder was set to 768, and the encoder layer number e_depth was set to 12. The vector dimension d_embed_dim in the decoder was set to 512, and the decoder layer number d_depth was set to 8.

According to the empirical evaluation results, *w* in Eq. (3) was set to 0.01. In Eq. (4), the value of $\gamma$ was set to 2, which was used to increase the discrimination of difficult samples. The initial value $\lambda _{0}$ of $\lambda$ in Eq. (5) was set to 0.01 to balance classification tasks and pose-estimation tasks.

### Experiment results

**Figure 6:**
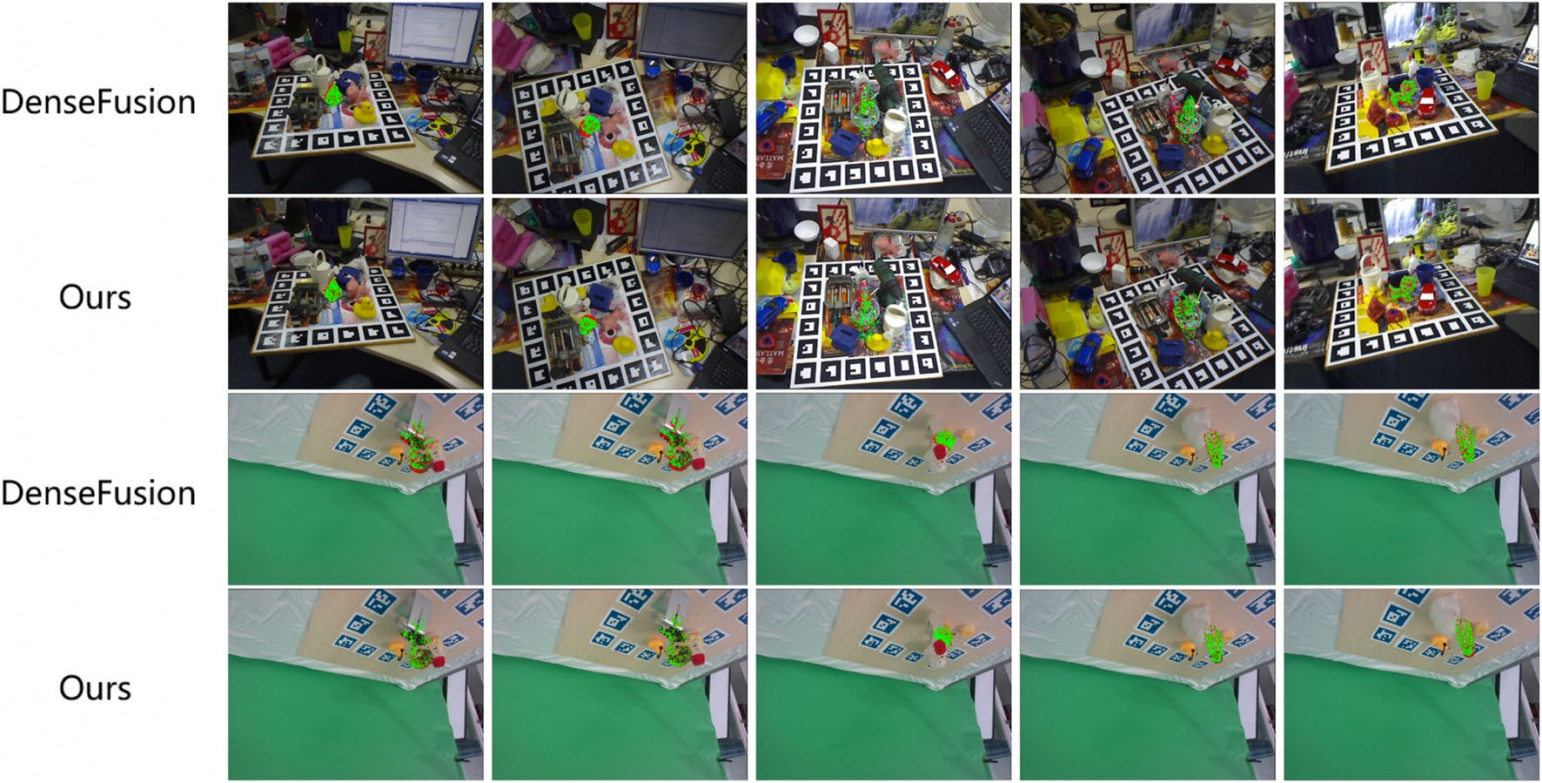
Visualization of predicted poses on the LineMOD (top two rows) and PMP (bottom two rows) datasets. The green point set represents the predicted object pose, whereas the red point set represents the ground truth object pose.

Herein, several experiments were conducted on the LineMOD public dataset and PMP dataset with the guarantee that the accuracy of our proposed method’s classification task reached 95%.

#### Performance on the LineMOD dataset

**Figure 7:**
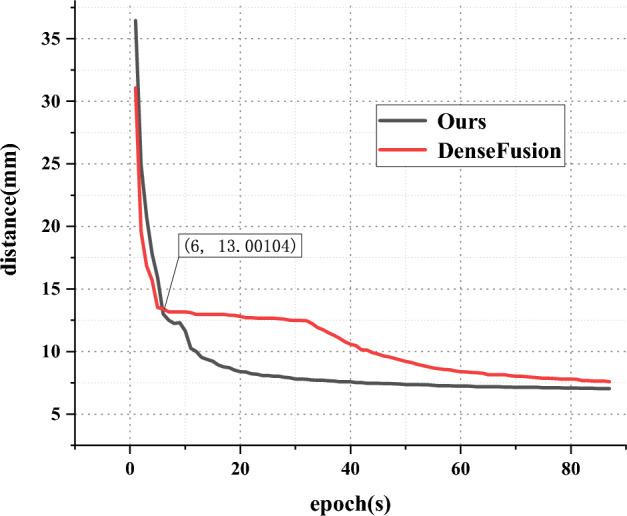
Rate of descent for ADD(-S) distance of our method and DenseFusion during training. The vertical axis represents the average distance between the calculated predicted value and real value, in mm. The line graph shows the results printed once per epoch.

Fig. 7 shows the comparison of the decline rate on the LineMOD dataset using the our pipeline and DenseFusion. Both models were trained for 87 epochs, and the test results were printed once per epoch. In our proposed method, the drop ratios of the classification and pose estimation task in the following experimental results were set to 0.3 and 0.1 respectively. The parameter settings are discussed in section 3.5.

Evidently from Fig. 7, starting from Epoch 6, our results has lower error distance than DenseFusion. Moreover, our proposed method exhibited substantially low trends in the first 20 epochs and began to decline steadily from Epoch 20. After 87 epochs of training, the minimum error distance reached 6.3 mm. By contrast, DenseFusion, owing to the lack of sufficient knowledge in the previous learning, gradually converged from Epoch 35, indicating that our method learned sufficient experiences in the early stage for pose estimation. The proposed method took 59 h to train 87 epochs, compared with 33 h required by DenseFusion, and our method achieved a distance of 11.64 mm at the 10th epoch, which is equivalent to the result of DenseFusion at Epoch 2. This indirectly shows that our method achieved results comparable to those of DenseFusion in less time upon gaining prior knowledge with the multi-task module.

**Table 2:** Evaluation results of each comparison model on the LineMOD dataset.

	SSD-6D+ICP	PointFusion	BB8	DenseFusion	Uni6D	Ours
Ape	65.0	70.4	96.6	89.0	93.7	93.8
Benchvise	80.0	80.7	90.1	92.6	99.8	94.5
Camera	78.0	60.8	86	95.2	95.9	**98.2**
Can	86.0	61.1	91.2	94.4	99.0	97.5
Cat	70.0	79.1	98.0	95.9	98.1	**98.1**
Driller	73.0	47.3	80.9	91.9	99.1	94.7
Duck	66.0	63.0	92.3	93.5	89.9	**93.5**
**Eggbox**	100.0	99.9	95.3	100.0	99.8	**100.0**
**Glue**	100.0	99.3	92.3	99.5	99.2	**99.8**
Holepuncher	49.0	71.8	95.3	92.5	90.2	94.7
Iron	78.0	83.2	84.8	96.0	99.4	98.2
Lamp	73.0	62.3	75.8	93.8	99.4	96.4
Phone	79.0	78.8	85.3	97.2	97.4	**97.8**
Mean	76.7	73.7	89.3	94.7	97.0	96.5
Extreme	37.0	35.9	23.0	9.0	9.9	**4.7**

$*$The bolded categories indicate symmetric objects.

The Mean value represents the average prediction accuracy for all objects, while the Extreme value represents the accuracy extreme for objects excluding symmetrical ones.

**Table 3:** Evaluation results of PMP dataset.

	DenseFusion	Ours
Boy	93.3	98.5
Duck	86.5	95.7
Glue	94.7	100.0
Roll*	96.1	100.0
Shelf	94.8	96.3
Mean	93.1	98.1
Extreme	8.3	4.3

$^*$the bolded categories indicate symmetric objects.

The final predicted object poses on the LineMOD and PMP dataset are visualized in Fig. 6, where the green and red pointsets display the 3D point cloud obtained by the predicted pose $p_{pred}$ and the real value *p* in a two-dimensional image after 2D–3D conversion, respectively. Five poses for the three objects are visualized in the figure, and the predicted poses for DenseFusion and our proposed method are listed in that order. In the two sets of results, the average precision errors of the DenseFusion and our method for estimating the object pose on the public dataset LineMOD are 3.0 mm and 2.1 mm, respectively, and the average errors on the collected datasets are 7.5 mm and 6.9 mm. Compared with DenseFusion, our method achieves smaller errors on both datasets.

#### Final results

The results of our method using ADD(-S) metric and^2,16,20–22^ are presented in Table 2 for the LineMOD dataset comparison, where SSD-6D^20^ uses the ICP algorithm for 3D point cloud conversion, BB8^2^ and DenseFusion use neural networks for overall refinement, and this study used the 10% of the object’s diameter as a threshold value. Five sets of experiments were conducted on the LineMOD dataset using the proposed method, listed in the table are the average results of five experiments; evidently, optimal results were achieved on 6 of the 13 objects. In the comparison of the final results, except for the symmetrical objects *eggbox* and *glue*, they all use ADD-S as the evaluation metric, which can get a higher accuracy, the asymmetrical objects with the lowest average accuracy are *ape* and *duck*, which both have the smallest diameters. The object which is asymmetrical with the highest accuracy rate obtained by the proposed method is *iron*, with an accuracy of 98.2%, and object with the lowest rate is *duck*, which has an accuracy of 93.5%, and the gap between them is only 4.7%. However, compared with SSD-6D^20^, PointFusion^21^, BB8^2^, DenseFusion^16^ and Uni6D^22^, the extreme values of accuracy they obtained were 37%, 35.9%, 23%, 9%, 9.9% respectively.

The above results show that the proposed method can reduce the accuracy deviation between objects while improving the accuracy rate, so that the estimation accuracy of the pose of objects with different shapes can be balanced. Among the five smallest-sized objects in the LineMOD dataset, namely ape, duck, cat, camera, and holepuncher, the proposed method achieves the best results for the first four objects, with accuracies of 93.8%, 93.5%, 98.1%, and 98.2%, respectively. Additionally, compared to DenseFusion, our method exhibits a 2.2% improvement for the holepuncher object. In Table 2, Uni6D^22^ achieved a fused RGBD input in the preprocessing stage by physical fusion, resulting in a prediction accuracy of 97.0%. However, when dealing with small-sized things as apes, ducks, and cats, the physical fusion input resulted in inefficient feature extraction inside the modality, leading to feature loss. Our solution lowered the accuracy bias from 9.9 to 4.7%, more than half as much as Uni6D. Table 3 presents our results of the five object tests in the PMP dataset, the proposed method was able to predict the small-size object poses well, essentially achieving an accuracy of 98.1%, while DenseFusion has an accuracy of 93.1%. The extreme value of the proposed method is only 4.3%, compared with 8.3% generated by DenseFusion.

### Two dropout rates

**Figure 8:**
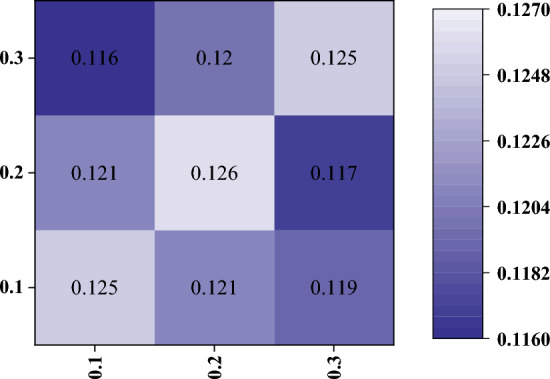
Different dropout rate combinations in our proposed method. The numbers in the figure represent the average error distance obtained by the model test, and the horizontal and vertical coordinates represent the dropout rate of the pose-estimation and classification tasks, respectively.

The effect of the $Drop=\left\{ D_{pose}, D_{cls}\right\}$ ratio on the LineMOD dataset of pose estimation was tested. Two drop ratios, ranging from $\left\{ 0.1, 0.2, 0.3\right\}$, were employed as the ratio of dropped neurons for classification tasks and pose-estimation tasks. To verify the influence of prior knowledge brought by parameter sharing on pose estimation, the error distance between the test and real values obtained by training for 10 epochs was recorded and expressed in the form of a heat map, as shown in the Fig. 8. The maximum distance in the figure was 12.6 mm. After evaluating, when the drop ratio was $\left\{ 0.0, 0.0\right\}$, the average distance obtained by the model was 12.7 mm, whereas the distance obtained by the DenseFusion model after the same 10 epoch test was 13.0 mm. Parameter sharing based on sparse sharing has its role in pose-estimation tasks.

Evidently from the Fig. 8, when the proportions of the two groups of drops were the same, that is, in the form of $\left\{ 0.1, 0.1\right\} , \left\{ 0.2, 0.2\right\} , \left\{ 0.3, 0.3\right\}$, it has greater error than those whose values are not equal, thus indicating that setting the same drop ratio for tasks with different levels of difficulties is inappropriate. The optimal set of results of the model was $\left\{ 0.1, 0.3\right\}$, which illustrates simpler classification tasks use fewer neurons. On the contrary, providing more parameters to the pose-estimation task can yield better results.

### Instance-level robotic grasping

Several duck items of the same size as in the PMP dataset were chosen for grasping experiments to test the efficacy of the proposed technique. Our experimental procedures included target localization and instance segmentation, target mask construction, and posture estimation to achieve end-to-end object grabbing. In the experiment, firstly, real-time picture data were fed into SegNet^23^, the pre-trained image segmentation network, to locate the position of the object and produce a mask image of the region. The following phase involved displaying the position discovered in the previous step as a white mask in the photograph, then estimating the pose of the target object and performing a grabbing experiment with the UR5 robotic arm. The following is a link to the video of the real-time robotic grasping experiment https://www.youtube.com/watch?v=xRtFaYIFpb4.

## Conclusion

In this work, we propose a multi-task parameter sharing method to improve the accuracy of pose estimation. The proposed method improves the accuracy in the field of pose estimation and narrows the deviation of the accuracy rate between objects of different shapes and sizes by using auxiliary tasks as prior information. More importantly, the proposed method can achieve a lower distance error than DenseFusion after fewer training times on the LineMOD dataset, and in the real robot arm grasping experiment, it can achieve the effect of real-time prediction of grasping. The proposed method works only for most rigid objects with regular geometry, and the accuracy decreases for objects with complex shapes or transparency, or under non-ideal conditions such as occlusion, and lighting changes.

## Data Availability

The LineMOD dataset analyzed for this study can be found at https://campar.in.tum.de/Main/StefanHinterstoisser in Ref .^16^. The PMP dataset used in this paper can be found at https://drive.google.com/file/d/1mYAT0baZj9VEPVOWs4YvCvo6hS59APvn/view?usp=drive_link. We will consider releasing the dataset generation technique, usable code, and comprehensive instructions for generating the PMP dataset in the future.
